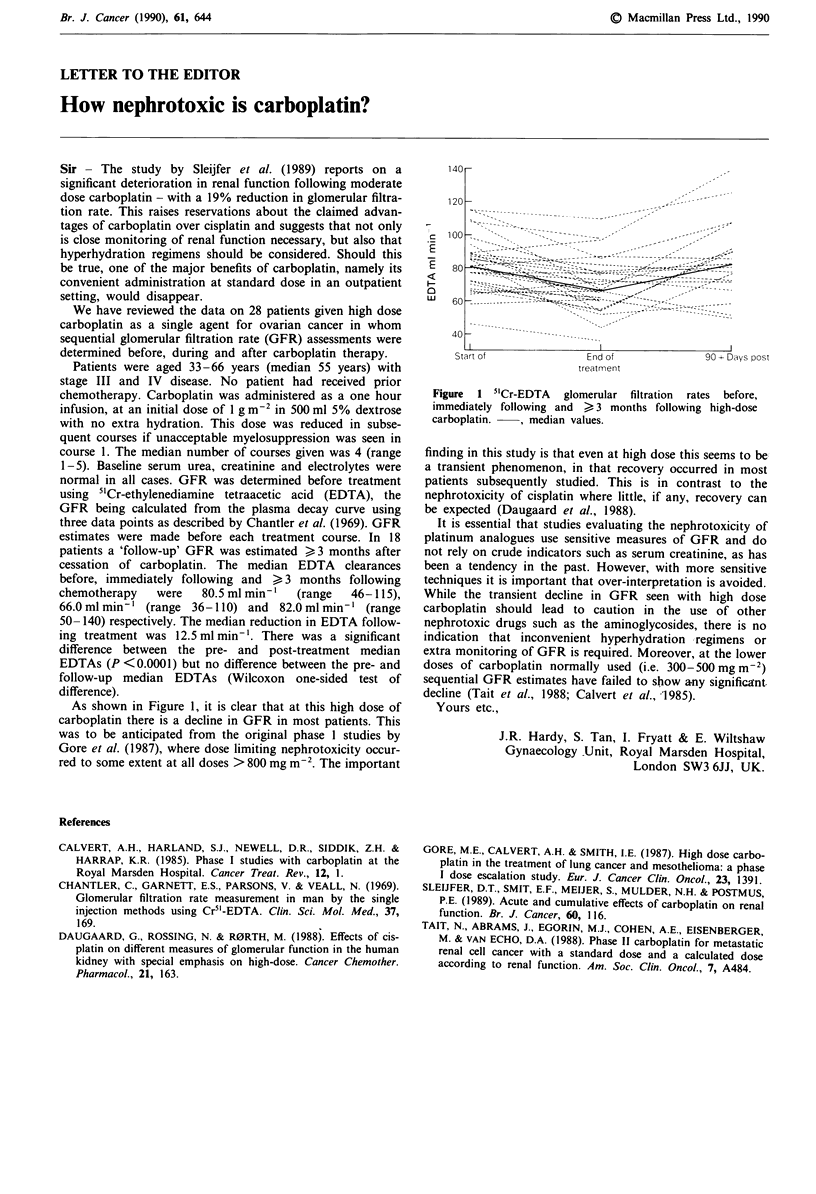# How nephrotoxic is carboplatin?

**DOI:** 10.1038/bjc.1990.143

**Published:** 1990-04

**Authors:** J. R. Hardy, S. Tan, I. Fryatt, E. Wiltshaw


Br. J. Cancer (1990), 61, 644 © Macmillan Press Ltd., 1990

### LETTER TO THE EDITOR

### How nephrotoxic is carboplatin?

**Sir** – The study by Sleijfer *et al.* (1989) reports on a significant deterioration in renal function following moderate dose carboplatin – with a 19% reduction in glomerular filtration rate. This raises reservations about the claimed advantages of carboplatin over cisplatin and suggests that not only is close monitoring of renal function necessary, but also that hyperhydration regimens should be considered. Should this be true, one of the major benefits of carboplatin, namely its convenient administration at standard dose in an outpatient setting, would disappear.

We have reviewed the data on 28 patients given high dose carboplatin as a single agent for ovarian cancer in whom sequential glomerular filtration rate (GFR) assessments were determined before, during and after carboplatin therapy.

Patients were aged 33–66 years (median 55 years) with stage III and IV disease. No patient had received prior chemotherapy. Carboplatin was administered as a one hour infusion, at an initial dose of 1 $g\,m^{-2}$ in 500 ml 5% dextrose with no extra hydration. This dose was reduced in subsequent courses if unacceptable myelosuppression was seen in course 1. The median number of courses given was 4 (range 1–5). Baseline serum urea, creatinine and electrolytes were normal in all cases. GFR was determined before treatment using $^{51}$Cr-ethylenediamine tetraacetic acid (EDTA), the GFR being calculated from the plasma decay curve using three data points as described by Chantler *et al.* (1969). GFR estimates were made before each treatment course. In 18 patients a 'follow-up' GFR was estimated $\geqslant 3$ months after cessation of carboplatin. The median EDTA clearances before, immediately following and $\geqslant 3$ months following chemotherapy were 80.5 ml $min^{-1}$ (range 46–115), 66.0 ml $min^{-1}$ (range 36–110) and 82.0 ml $min^{-1}$ (range 50–140) respectively. The median reduction in EDTA following treatment was 12.5 ml $min^{-1}$. There was a significant difference between the pre- and post-treatment median EDTAs ($P < 0.0001$) but no difference between the pre- and follow-up median EDTAs (Wilcoxon one-sided test of difference).

As shown in Figure 1, it is clear that at this high dose of carboplatin there is a decline in GFR in most patients. This was to be anticipated from the original phase 1 studies by Gore *et al.* (1987), where dose limiting nephrotoxicity occurred to some extent at all doses $> 800$ mg $m^{-2}$. The important finding in this study is that even at high dose this seems to be a transient phenomenon, in that recovery occurred in most patients subsequently studied. This is in contrast to the nephrotoxicity of cisplatin where little, if any, recovery can be expected (Daugaard *et al.*, 1988).

**Figure 1** $^{51}$Cr-EDTA glomerular filtration rates before, immediately following and $\geqslant 3$ months following high-dose carboplatin. ——, median values.

It is essential that studies evaluating the nephrotoxicity of platinum analogues use sensitive measures of GFR and do not rely on crude indicators such as serum creatinine, as has been a tendency in the past. However, with more sensitive techniques it is important that over-interpretation is avoided. While the transient decline in GFR seen with high dose carboplatin should lead to caution in the use of other nephrotoxic drugs such as the aminoglycosides, there is no indication that inconvenient hyperhydration regimens or extra monitoring of GFR is required. Moreover, at the lower doses of carboplatin normally used (i.e. 300–500 mg $m^{-2}$) sequential GFR estimates have failed to show any significant decline (Tait *et al.*, 1988; Calvert *et al.*, 1985).

Yours etc.,

J.R. Hardy, S. Tan, I. Fryatt & E. Wiltshaw
Gynaecology Unit, Royal Marsden Hospital,
London SW3 6JJ, UK.

**References**

CALVERT, A.H., HARLAND, S.J., NEWELL, D.R., SIDDIK, Z.H. & HARRAP, K.R. (1985). Phase I studies with carboplatin at the Royal Marsden Hospital. *Cancer Treat. Rev.*, **12,** 1.

CHANTLER, C., GARNETT, E.S., PARSONS, V. & VEALL, N. (1969). Glomerular filtration rate measurement in man by the single injection methods using Cr$^{51}$-EDTA. *Clin. Sci. Mol. Med.*, **37,** 169.

DAUGAARD, G., ROSSING, N. & RØRTH, M. (1988). Effects of cisplatin on different measures of glomerular function in the human kidney with special emphasis on high-dose. *Cancer Chemother. Pharmacol.*, **21,** 163.

GORE, M.E., CALVERT, A.H. & SMITH, I.E. (1987). High dose carboplatin in the treatment of lung cancer and mesothelioma: a phase I dose escalation study. *Eur. J. Cancer Clin. Oncol.*, **23,** 1391.

SLEIJFER, D.T., SMIT, E.F., MEIJER, S., MULDER, N.H. & POSTMUS, P.E. (1989). Acute and cumulative effects of carboplatin on renal function. *Br. J. Cancer*, **60,** 116.

TAIT, N., ABRAMS, J., EGORIN, M.J., COHEN, A.E., EISENBERGER, M. & VAN ECHO, D.A. (1988). Phase II carboplatin for metastatic renal cell cancer with a standard dose and a calculated dose according to renal function. *Am. Soc. Clin. Oncol.*, **7,** A484.